# Experience with International Neuroblastoma Staging System and Pathology Classification

**DOI:** 10.1038/sj.bjc.6600231

**Published:** 2002-04-08

**Authors:** H Ikeda, T Iehara, Y Tsuchida, M Kaneko, J Hata, H Naito, M Iwafuchi, N Ohnuma, H Mugishima, Y Toyoda, M Hamazaki, J Mimaya, S Kondo, K Kawa, A Okada, E Hiyama, S Suita, H Takamatsu

**Affiliations:** Department of Pediatric Surgery, Dokkyo University School of Medicine, Koshigaya Hospital, 2-1-50 Minami-Koshigaya, Koshigaya, Saitama 343-8555, Japan; Department of Pediatrics, Kyoto Prefectural University of Medicine, Hirokohji, Kawaramachi-dori, Kamigyo-ku, Kyoto 602-8566, Japan; Study Group of Neuroblastoma, Gunma Children's Medical Center, 377 Shimohakoda, Hokkitsu, Seta-gun, Gunma 377-8577, Japan; Department of Pediatric Surgery, University of Tsukuba, 1-1-1 Tennodai, Tsukuba 305-8575, Japan

**Keywords:** neuroblastoma, International Neuroblastoma Staging System (INSS), International Neuroblastoma Pathology Classification (INPC), *MYCN* amplification, DNA ploidy, 1p deletion

## Abstract

The International Neuroblastoma Staging System and Pathology Classification were proposed in 1988 and in 1999, respectively, but their clinical value has not yet been fully studied in new patients. Six hundred and forty-four patients with neuroblastoma treated between January 1995 and December 1999 were analysed by these classifications. The 4-year overall survival rate of patients <12 months of age with INSS stages 1, 2A, 2B, 3 and 4S disease was 98.5%, which was significantly higher than the 73.1% rate in stage 4 patients <12 months (*P*<0.0001). When patients were ⩾12 months, the 4-year overall survival rate of patients with neuroblastoma at 1, 2A, 2B and 3 stages was 100% and that of patients at stage 4 was 48.5% (*P*<0.0001). As to the International Neuroblastoma Pathology Classification histology, the 4-year overall survival rate was 98.8% in patients with favourable histology and 60.7% in those with unfavourable histology in the <12 months group (*P*<0.0001). In the ⩾12 months group, the 4-year oral survival of patients with favourable histology was 95.3% and that of patients with unfavourable histology was 50.6% (*P*<0.0001). Among biological factors, *MYCN* amplification, DNA diploidy and 1p deletions were significantly associated with poor prognosis in patients <12 months, as were *MYCN* amplification and DNA diploidy in patients ⩾12 months of age. Multivariate analysis showed that the INSS stage (stage 4 *vs* other stages) and International Neuroblastoma Pathology Classification histology (unfavourable *vs* favourable) were significantly and independently associated with the survival of patients undergoing treatment, stratified by age, stage and *MYCN* amplification (*P*=0.0002 and *P*=0.0051, respectively).

*British Journal of Cancer* (2002) **86**, 1110–1116. DOI: 10.1038/sj/bjc/6600231 www.bjcancer.com

© 2002 Cancer Research UK

## 

International cooperative groups have developed new classifications of neuroblastoma risk groups to compare treatment results internationally and seek effective means to deal with neuroblastoma, particularly in advanced stages. The International Neuroblastoma Staging System (INSS) was originally proposed in 1988, and revised in 1993 (Brodeur *et al*, 1993). Since then the INSS has been used worldwide (Castleberry *et al*, 1994; Kaneko *et al*, 1998), replacing the previous staging systems of different groups. The International Neuroblastoma Pathology Classification (INPC) that is fundamentally based on Shimada's classification was also proposed in 1999 to provide neuroblastoma study groups with a common language (Shimada *et al*, 1999). However, the clinical importance of these classifications has never been extensively evaluated; Castleberry *et al* (1994) applied the INSS only retrospectively for their patients treated between 1981 and 1990. Since the predictability of prognostic factors often depends on the intensity or efficacy of treatment, it is important to examine the value of these risk classifications in a study in which highly effective modern treatments are applied (Kawa *et al*, 1999; Matthay *et al*, 1999).

Clinical results achieved prior to 1991 by us and by others (Brodeur *et al*, 1984; Seeger *et al*, 1985; Sawaguchi *et al*, 1990) clearly showed that patients with *MYCN-*amplified tumours have a worse prognosis than those without. It was therefore decided in Japan in 1991 to administer intensive induction chemotherapy with a double dose of cyclophosphamide to high-risk patients with *MYCN* amplification (Kaneko *et al*, 1998), and that strategy resulted in improved clinical results (Kawa *et al*, 1999). In the present study we examined the prognostic value of the INSS, the INPC and biological factors including *MYCN* amplification, DNA ploidy and 1p deletion in patients with neuroblastoma who underwent treatment between 1995 and 1999.

## PATIENTS AND METHODS

A total of 731 patients with newly diagnosed neuroblastoma whose treatment was started between January 1995 and December 1999 were retrospectively reviewed. Patients ⩾12 months of age were treated with the protocols for advanced neuroblastoma in which treatment was stratified by stage and *MYCN* amplification status (Kaneko *et al*, 1998). Stem cell transplantation was performed in 65.4% of stage 4 patients and in 67.4% of *MYCN*-amplified patients ⩾12 months of age. Patients <12 months of age were registered with the Japanese Infantile Neuroblastoma Cooperative Study and were treated with the protocols for infant neuroblastoma (Matsumura and Michon, 2000). Briefly, these infant patients were first examined for the presence or absence of *MYCN* amplification, and those without amplification were treated with or without chemotherapy based on the INSS stage. Patients with *MYCN* amplification were treated, like patients ⩾12 months of age, with combination chemotherapy with or without stem cell transplantation. Stem cell transplantation was carried out in 27.5% of stage 4 patients and in 41.7% of *MYCN*-amplified patients <12 months of age (Table 1

**Table 1:**
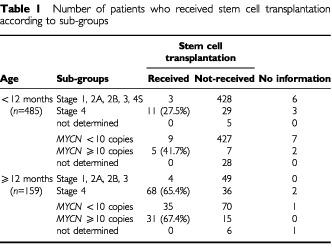
Number of patients who received stem cell transplantation according to sub-groups

).

Patient information with regard to age, gender, stage, histology and biological characteristics of the tumour including *MYCN* amplification, DNA ploidy and 1p deletion was collected from the participating institutions (Appendix). Two pathologists, one of whom was a member of the International Neuroblastoma Pathology Committee, reviewed all of the specimens according to the INPC (Shimada *et al*, 1999). The *MYCN* gene copy number was determined by Southern blot hybridisation, and amplification was defined as ⩾10 copies. DNA content analysis was performed by means of flow cytometry, and tumours were classified into diploid tumours (DNA index=1) and aneuploid tumours (DNA index ⩾1.1). No genes responsible for *MYCN* amplification have yet been identified and there is no consensus on the definition of prognostically significant deletions of 1p so that no single uniform method to detect 1p deletions could be used. The method was therefore left to the discretion of each laboratory, and 1p deletions were defined as present when karyotypic analysis showed large deletions in the distal region of 1p or when molecular analysis demonstrated small deletions of 1p36 by examining the loss of heterozygosity (LOH) with gene markers (Ohtsu *et al*, 1997).

Patient outcomes were followed up as of 30 September 2000, and the results were obtained in 675 patients. There were 31 patients with screening-detected tumours who had been observed without histological confirmation and definitive treatment. Excluding these 31 patients because of insufficient data, 644 patients, 368 males and 276 females, were included in the final analyses. The age of the patients ranged from 0 to 221 months (median, 8 months), and 485 patients were <12 months of age with 159 patients ⩾12 months.

Differences between the two groups in categorical data were analysed by means of Fisher's exact probability test or the chi-square test. Overall survival was estimated by the Kaplan-Meier method and the difference between the curves was compared with the log-rank test. For multivariate analysis, the Cox regression models were used to identify independent prognostic factors. Statistical analysis was performed with SPSS 7.5J for Windows Medical Pack (SPSS Inc., Chicago, IL, USA) and a *P* value of <0.05 was considered statistically significant.

## RESULTS

### INSS, INPC and biological prognostic factors

The INSS stage, INPC histology and biological prognostic factors of the 644 patients are shown in Table 2

**Table 2:**
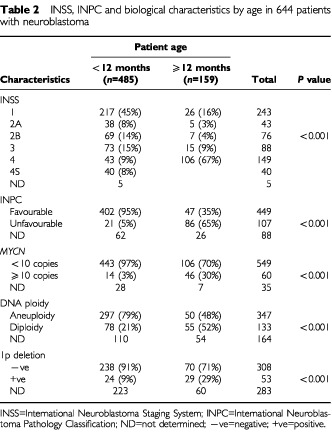
INSS, INPC and biological characteristics by age in 644 patients with neuroblastoma

. INSS stage was determined in 639 patients, and there was a significant difference in stage distribution between patients <12 months of age and those ⩾12 months (*P*<0.001). Among the 480 patients <12 months of age, 43 (9%) had stage 4 disease, and there were 106 (67%) stage 4 patients among the 159 patients ⩾12 months. Histology was evaluated according to the INPC in 556 patients. Of 423 patients <12 months of age, favourable histology was diagnosed in 402 patients (95%) and unfavourable histology in 21 patients (5%). Among the 133 patients ⩾12 months of age, 47 patients (35%) had favourable histology and 86 patients (65%) unfavourable histology. The difference between the two age groups in unfavourable histology was statistically significant (*P*<0.001).

The occurrence of unfavourable biological characteristics was also significantly different between <12 months and ⩾12 months of age. The *MYCN* gene copy number was examined in 609 patients. Greater than ⩾10 copies of *MYCN* were observed in 14 (3%) of 457 patients <12 months of age, and in 46 (30%) of 152 patients ⩾12 months (*P*<0.001). DNA content analysis by flow cytometry was carried out in 480 patients and 78 (21%) of 375 patients in the younger age group and 55 (52%) of 105 patients in the older age group had diploid tumours (*P*<0.001). Chromosomal abnormalities were examined in 361 patients, and the presence of 1p deletions was demonstrated in 24 (9%) of 262 patients <12 months of age and in 29 (29%) of 99 patients ⩾12 months (*P*<0.001).

### Overall survival rates

The median follow-up for all of the 644 patients was 1120 days, and that for living patients 1179 days. The 2- and 4-year overall survival (2-OS and 4-OS) rates of patients <12 months of age were 96.5 and 96.2%, respectively, and those of patients who were ⩾12 months of age were 77.6 and 64.6%, respectively. Seventy-five per cent of the patients in this series were <12 months of age. To avoid the bias relating to the patient age distribution, the following data analyses were performed in two groups of <12 months and ⩾12 months of age.

The 2-OS and 4-OS rates of the 639 patients are shown according to the INSS stage in Table 3

**Table 3:**
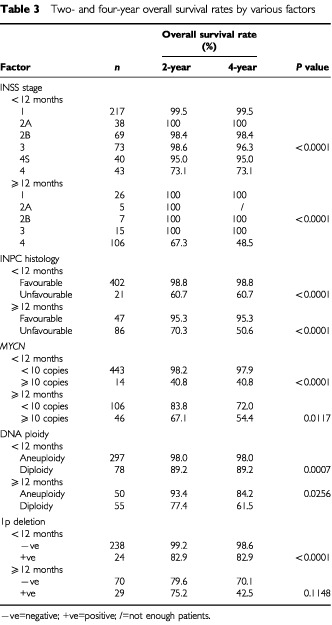
Two- and four-year overall survival rates by various factors

. There were no differences in the overall survival rates among stages 1, 2A, 2B, 3 and 4S patients <12 months of age, and none among stages 1, 2A, 2B and 3 patients ⩾12 months of age, either. Therefore, infants with stages 1, 2A, 2B, 3 and 4S disease in the younger age group, and patients with stages 1, 2A, 2B and 3 disease in the older age group were combined in the subsequent survival analyses.

**Figure 1:**
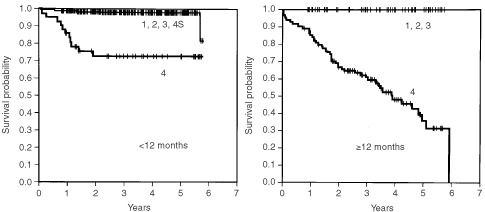
Cumulated overall survival rates are shown. In patients <12 months of age, the 2- and 4-year overall survival rates for patients in stages 1, 2A, 2B, 3 and 4S combined (*n*=437) were 98.8 and 98.5%, respectively, which were significantly higher than the 73.1 and 73.1% for patients in stage 4 (*n*=43) (*P*<0.0001). In patients ⩾12 months of age, the 2- and 4-OS rates for patients in 1, 2A, 2B and 3 stages combined (*n*=53) were 100 and 100%, while those for patients in stage 4 (*n*=106) were 67.3 and 48.5%, respectively (*P*<0.0001).

In patients <12 months of age, the 4-OS rate of patients in stages 1, 2A, 2B, 3 and 4S combined was 98.5%, which was significantly higher than the 73.1% of patients in stage 4 (*P*<0.0001). In patients ⩾12 months of age, the 4-OS rate of patients in stages 1, 2A, 2B and 3 combined was 100%, whereas that of patients with stage 4 disease was 48.5%; the difference was statistically significant (*P*<0.0001) (Figure 1

**Figure 5:**
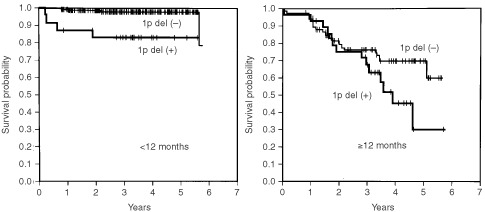
Cumulated overall survival rates for patients <12 months of age and ⩾12 months of age are shown. Among patients <12 months of age, the 2- and 4-year overall survival rates for patients without 1p deletion (*n*=238) were 99.2 and 98.6%, respectively, which were significantly higher than the 82.9 and 82.9% for patients with 1p deletion (*n*=24) (*P*<0.0001). In patients ⩾12 months of age, the 2- and 4-OS rates for patients without 1p deletion (*n*=70) were 79.6 and 70.1%, respectively, whereas those for patients with it (*n*=29) were 75.2 and 42.5%, respectively (*P*=0.1148).

**Figure 4:**
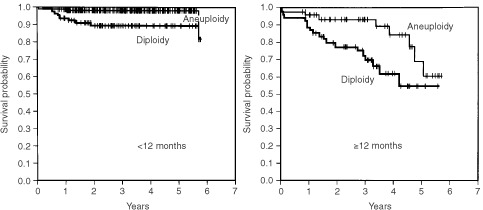
Cumulated overall survival rates for patients <12 months of age and ⩾12 months of age are shown. Among patients <12 months of age, the 2- and 4-year overall survival rates for patients with aneuploidy (*n*=297) were 98.0 and 98.0%, respectively, which were significantly higher than the 89.2 and 89.2% for patients with diploidy (*n*=78) (*P*=0.0007). In patients ⩾12 months of age, the 2- and 4-OS rates for patients with aneuploidy (*n*=50) were 93.4 and 84.2%, respectively, while those for patients with diploidy (*n*=55) were 77.4 and 61.5%, respectively (*P*=0.0256).

**Figure 3:**
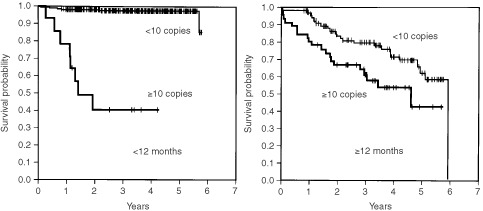
Cumulated overall survival rates for patients <12 months of age and ⩾12 months of age are shown. Among patients <12 months of age, the 2- and 4-year overall survival rates for patients with <10 copies of *MYCN* (*n*=443) were 98.2 and 97.9%, respectively, which were significantly higher than the 40.8 and 40.8% for patients with ⩾10 copies of *MYCN* (*n*=14) (*P*<0.0001). In patients ⩾12 months of age, the 2- and 4-OS rates for patients with <10 copies of *MYCN* (*n*=106) were 83.8 and 72.0%, respectively, whereas those of patients with ⩾10 copies of *MYCN* (*n*=46) were 67.1 and 54.4%, respectively (*P*=0.0117).

).

**Figure 2:**
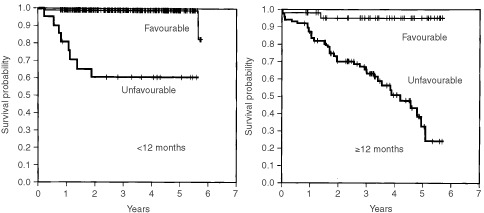
Cumulated overall survival rates for patients <12 months of age and ⩾12 months of age are shown. The 2- and 4-OS rates for patients with favourable histology (*n*=402) were 98.8 and 98.8%, respectively, significantly higher than the rates of 60.7 and 60.7% for patients with unfavourable histology (*n*=21) when the patients were <12 months of age (*P*<0.0001). The 2- and 4-OS rates for patients with favourable histology (*n*=47) were 95.3 and 95.3%, respectively, significantly higher than the corresponding rates of 70.3 and 50.6% for patients with unfavourable histology (*n*=86) in patients ⩾12 months of age (*P*<0.0001).

INPC histology was also shown to be a significant prognostic factor. The 4-OS rate of patients with favourable histology was 98.8%, significantly higher than the rate of 60.7% for patients with unfavourable histology when the patients were <12 months of age (*P*<0.0001) (Figure 2

). The survival rate for patients with favourable histology was 95.3%, significantly higher than the 50.6% rate for patients with unfavourable histology in patients ⩾12 months of age (*P*<0.0001).

Among patients <12 months of age, overall survival rates for patients with favourable biological characteristics (<10 copies of the *MYCN* gene, aneuploidy and absence of 1p deletions) were significantly higher than those for patients with unfavourable characteristics (⩾10 copies of the *MYCN* gene, diploidy and the presence of 1p deletions) (Figures 3, 4 and 5, respectively) (*P*<0.0001, *P*=0.0007 and *P*<0.0001, respectively). Of the 14 patients with *MYCN* amplification, eight patients had died before this retrospective review, and the 4-OS rate was 40.8%.

When the patients were ⩾12 months of age, *MYCN* amplification and DNA diploidy were significantly associated with poor prognosis. The 2-OS and 4-OS rates for patients with neuroblastoma with amplified *MYCN* were 67.1 and 54.4%, respectively, and significantly lower than the 83.8 and 72.0% for patients without *MYCN* amplification (*P*=0.0117). The 2-OS and 4-OS rates for patients with diploid tumour were 77.4 and 61.5%, respectively, which were significantly lower than the 93.4 and 84.2%, respectively, for patients with aneuploid tumour (*P*=0.0256). In the overall survival rate there was no significant difference between patients with and without 1p deletion (*P*=0.1148).

### Multivariate analysis of prognostic factors

A multivariate analysis with Cox regression models was undertaken in 460 patients with complete data including age, INSS stage, INPC histology, *MYCN* amplification and DNA ploidy (Table 4

**Table 4:**
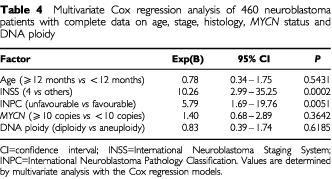
Multivariate Cox regression analysis of 460 neuroblastoma patients with complete data on age, stage, histology, *MYCN* status and DNA ploidy

). 1p deletion was excluded from the analysis because genetic abnormalities of chromosome 1p were examined in only 361 (56.1%) of 644 patients. The analysis showed that INSS stage and INPC histology were significantly and independently associated with patient survival (*P*=0.0002 and *P*=0.0051, respectively), and that the biological variables, *MYCN* amplification and DNA ploidy, did not have prognostic significance after correcting for stage and histology by the INSS and INPC.

## DISCUSSION

A protocol with stratification of treatment mainly based on the presence or absence of *MYCN* amplification was introduced in our group study in 1991 (Kaneko *et al*, 1998). This protocol was designed to administer intensive induction chemotherapy with a double dose of cyclophosphamide and to do stem cell transplantation to high-risk patients with *MYCN* amplification, and analysis of outcomes showed a significantly higher response rate and a number of long-term survivors in patients with *MYCN* amplification (Kawa *et al*, 1999). For infantile neuroblastomas, another group study was started in 1994, and the protocol was revised in 1998 (Matsumura and Michon, 2000).

The INSS is a surgicopathologic staging system and the chief difference between it and the Evans staging system is the definition of stage of locoregional disease (Evans *et al*, 1971; Brodeur *et al*, 1993), but the present study showed that there was no significant difference in survival rate between the stages of localised disease. The 4-OS rate for patients <12 months of age with stages 1, 2A, 2B, 3 and 4S disease was 98.5% and that for patients ⩾12 months with stages 1, 2A, 2B and 3 disease was 100% (Table 3). On the other hand, the survival rate in patients with metastatic disease (excluding stage 4S) was significantly worse than in patients with non-metastatic disease, as reported by others (Hero *et al*, 2000). Previously, differences in survival rates among individual stages were clear (Breslow and McCann, 1971; Matthay *et al*, 1998). Breslow and McCann (1971) demonstrated that the prognosis for children with neuroblastoma becomes worse in the order stage I, stage IVS, stage II, stage III and stage IV, with coefficients of 1.684, 0.661, 0.431, −0.092 and −2.684, respectively. Matthay *et al* (1998) found that the survival rate for stage III patients ⩾12 months of age treated between 1991 and 1995 was still worse than for stage III patients <12 months of age (*P*=0.01). Our follow-up period is shorter than in other studies, and the number of patients in the older age group was rather small; especially there being only 15 patients ⩾12 months of age with stage 3 disease in the present study, which indicated that there was no difference in the survival rate between stage 3 disease and combined stages 1, 2A and 2B disease in patients ⩾12 months of age treated between 1995 and 1999. Among these 15 patients ⩾12 months of age in stage 3, there were five patients with unfavourable INPC and two patients with *MYCN* amplification. One patient was surviving for 33 months with disease but the remaining 14 were disease free. We therefore would like to consider that this implies some progress in treatment.

The INPC (Shimada *et al*, 1999) distinguished prognostic groups clearly. Previous studies showed that the histological group provides additional prognostic information beyond factors such as age, stage and *MYCN* oncogene status (Suita *et al*, 1994; Rubie *et al*, 1997; Matthay *et al*, 1998). The results of this study indicate that INPC histology is a powerful and independent predictor of prognosis.

*MYCN* amplification in neuroblastoma is associated with advanced stage, rapid tumour progression and poor prognosis (Seeger *et al*, 1985; Suita *et al*, 1994; Combaret *et al*, 1996). Nevertheless, there is controversy over the prognostic importance of *MYCN* in localised neuroblastoma. For example, it was shown that patients who have stage 2 neuroblastoma with *MYCN* amplification are at higher risk than those without it (Rubie *et al*, 1997; Alvarado *et al*, 2000; Perez *et al*, 2000), but another study demonstrated that the presence of *MYCN* amplification in localised neuroblastoma does not necessarily indicate an adverse outcome (Cohn *et al*, 1995). In the present study, the presence of *MYCN* amplification was a significant predictor of poor prognosis both in patients <12 months and ⩾12 months of age. Eight of 14 patients with *MYCN* amplification died within 2 years after diagnosis in the group of patients <12 months of age, and the 4-OS rate of patients with *MYCN* amplification was only 54.4% in the patients ⩾12 months of age, whereas the 4-OS rate was 72.0% in patients without *MYCN* amplification in the same age group. Nevertheless, multivariate analysis showed that *MYCN* amplification is not an independent prognostic factor. The lack of significance of *MYCN* amplification as an independent prognostic factor is certainly associated with improved survival for patients with *MYCN* amplification that was achieved by stratification of treatment with high-dose chemotherapy and blood stem cell transplantation (Kawa *et al*, 1999). It shows, perhaps, that there was some success with the treatment approach, not that *MYCN* has lost importance as a prognostic factor.

DNA ploidy has been considered to be another significant prognostic predictor (Look *et al*, 1991), but the difference in survival between the patients with diploid tumours and those with aneuploid tumours was not great. The 4-OS rates in patients with aneuploid tumours and with diploid tumours were 98.0 and 89.2%, respectively, among patients <12 months of age, and 84.2 and 61.5%, respectively, in patients ⩾12 months of age. These results appear to support the findings of Look and associates (Look *et al*, 1991) that demonstrated a close correlation between tumour ploidy and outcome in a large series of patients <24 months of age, but not in older patients with metastatic neuroblastoma.

1p deletion was a significant predictor of prognosis in our patients <12 months of age, but not in patients ⩾12 months. This result is comparable to the results from a study (Maris *et al*, 2000) in which LOH at 1p36 predicted progression free survival, but not decreased overall survival in patients with single copy of *MYCN*. Tumour suppressor genes responsible for the development of neuroblastoma are assumed to be present on chromosome 1p36, but no conclusive results on the identification and location of such genes for LOH analysis have yet been reported. In this study, 1p deletion was defined when either karyotype analysis or screening for 1p36 LOH (Ohtsu *et al*, 1997) showed such deletions as described above. Important genetic abnormalities might not have been detected, which in turn would have affected survival analysis.

A number of factors that correlate with the response to treatment and outcome of patients with neuroblastoma have been identified, including patient age, stage, histology, *MYCN* amplification, DNA ploidy, 1p deletions, 17q gain (Meddeb *et al*, 1996; Bown *et al*, 1999), *TRKA* expression (Nakagawara *et al*, 1993; Suzuki *et al*, 1993) and CD44 cell-surface expression (Combaret *et al*, 1996). This study evaluated the prognostic importance of the first six factors that are generally considered essential in decision making for treatment, and found that INSS and INPC were significant and independent predictors of prognosis in patients who underwent stratification of treatment. The study included not only patients with clinically detected neuroblastoma but also those identified by mass screening. In Japan, nearly 70% of neuroblastomas are detected by screening, usually at the age of 6 to 9 months (Tsuchida *et al*, 2000), hence the high incidence of infantile neuroblastoma in the present study, and the data were analysed thoroughly in the two age groups.

## Appendix

### Participating institutions chief investigators

Gunma Children's Medical Center, Gunma (Tsuchida Y, Kuroiwa M); Kyoto Prefectural University of Medicine, Kyoto (Iehara T, Sugimoto T, Sawada T); University of Tsukuba, Tsukuba (Kaneko M); Keio University, Tokyo (Hata J); Dokkyo University, Koshigaya (Ikeda H); National Sapporo Hospital, Sapporo (Naito H); Hokkaido University, Sapporo (Sasaki F); Yamagata University, Yamagata (Mitsui T); Fukushima Medical University, Fukushima (Kikuta A); Niigata University, Niigata (Iwafuchi M, Kaneda S); Ibaraki Children's Hospital, Mito (Kenmotsu H); Jichi Medical School, Tochigi (Itonaga N); Saitama Children's Medical Center, Iwatsuki (Yamamoto K), Saitama Medical School, Saitama (Shibuya A); National Defense Medical College, Tokorozawa (Sekine I); Chiba University, Chiba (Ohnuma N, Matsunaga M); Chiba Children's Hospital, Chiba (Etoh T); Nihon University, Tokyo (Mugishima H); University of Tokyo, Tokyo (Kobayashi M); National Children's Hospital, Tokyo (Kumagai M); Juntendo University, Tokyo (Fujita H); Showa University, Tokyo (Tanaka D); Jikei University, Tokyo (Yamazaki Y); St. Luke's International Hospital, Tokyo (Hosoya R); Kiyose Metropolitan Children's Hospital, Kiyose (Hayashi A, Hirobe S); Hachioji Metropolitan Children's Hospital, Hachioji (Nishina T); Kanagawa Children's Medical Center, Yokohama (Toyoda Y); St. Marianna University, Kawasaki (Nakada K); Kitasato University, Sagamihara (Nakadate H); Tokai University, Isehara (Yokoyama S); Shizuoka Children's Hospital, Shizuoka (Hamazaki M, Mimaya J, Horigoshi Y); Gifu City Hospital, Gifu (Takao A); Kanazawa Medical University, Ishikawa (Kawano M); Fukui Medical University, Fukui (Yazawa A); Nagoya City University, Nagoya (Kondo S); Mie University, Tsu (Komada Y, Hirayama M, Hori H); Shiga University of Medical Science, Ohtsu (Ohta S); Osaka Medical Center for Maternal and Child Health, Izumi (Kawa K, Inoue M, Yoneda M, Oue T); Osaka University, Suita (Okada A, Kusafuka T); Osaka City Medical Center, Osaka (Nakamura T); Kinki University, Sakai (Miyata H); Okayama University, Okayama (Nishiuchi R); Hiroshima University, Hiroshima (Hiyama E); National Kure Hospital, Kure (Tanaka T); Yamaguchi University, Ube (Ayukawa H); Tokuyama Hospital, Tokuyama (Uchida M); Kagawa Children's Hospital, Zentsuji (Iwai T); Ehime University, Ehime (Ishida Y); Ehime Prefectural Hospital, Matsuyama (Oofuji Y); Kochi Medical University, Nangoku (Wakiguchi H); Kochi Prefectural Hospital, Kochi (Yoshikawa K); Kyushu University, Fukuoka (Suita S); National Kyushu Cancer Center, Fukuoka (Okamura J); Saga Medical University, Saga (Miyazaki S, Koga H); Oita Medical University, Oita (Suenobu S); Miyazaki Medical University, Miyazaki (Kuroda H); Kagoshima University, Kagoshima (Takamatsu H, Fukushige T).
